# Reproductive health at conflict borders: a cross-sectional survey of human rights violations and perinatal outcomes at the Thai-Myanmar border

**DOI:** 10.1186/s13031-021-00347-8

**Published:** 2021-03-10

**Authors:** Stephanie M. Koning, Kaylee Scott, James H. Conway, Mari Palta

**Affiliations:** 1 Department of Population Health Sciences, University of Wisconsin School of Medicine and Public Health, Madison, WI USA; 2 Present address: Institute for Policy Research, Northwestern University, 2040 Sheridan Road, Evanston, IL 60208 USA; 3 Department of Pediatrics, University of Wisconsin School of Medicine and Public Health, Madison, WI USA

**Keywords:** Reproductive health, International health, Conflict, Displacement, Migration, Violence, Social epidemiology

## Abstract

**Background:**

Human rights violations (HRVs) are common in conflict and displacement contexts. Women are especially vulnerable to HRVs in these contexts, and perinatal health is acutely sensitive to related stressors and health care barriers. However, how HRVs affect immediate and long-term perinatal health in chronic displacement settings has not been closely investigated. Furthermore, it remains unclear whether and how HRVs in these contexts are tied directly to displacement circumstances or other marginalizing factors affecting local migrant and minority populations generally.

**Methods:**

We investigated these questions using novel survey data from 577 women at the northern Thai-Myanmar border, where thousands of people have fled conflict in Shan State, Myanmar, for refuge in a range of precarious settings in Thailand, including unofficial refugee camps, villages, and worksites. We compared HRV exposures by ethnicity, country of birth, legal documentation, and residential setting. We then analyzed perinatal outcomes associated with HRV frequency, timing, and type.

**Results:**

Birth in Myanmar, and ethnic minority and precarious legal status more broadly, predicted higher HRV prevalence. HRV frequency significantly predicted unmet antenatal care and lower birth weight, along with HRVs related to labor exploitation and violence or conflict. HRVs timed closer to pregnancies were more adversely associated with perinatal outcomes. Resource/property deprivation was the strongest predictor of pregnancy complications.

**Conclusions:**

Human rights must be urgently attended to, through expanded HRV screenings and responsive care, and policy changes to further protect migrant workers, displaced persons, and others in precarious legal status situations.

**Supplementary Information:**

The online version contains supplementary material available at 10.1186/s13031-021-00347-8.

## Introduction

Violence is a global health crisis [1, 2]. Armed conflict is escalating globally and has led to higher concentrations of displaced persons (DPs) at international borders [3]. This trend is a result of more frequent and prolonged armed conflicts occurring in civilian spaces, placing local populations at risk from violence and human rights violations (HRVs), including women and children [4, 5]. Thailand’s border with Myanmar offers an informative case study for understanding the impact of host settings on individuals affected by displacement. For over sixty years, millions of people have fled discrimination, persecution, violence, poverty, and political failures in Myanmar to neighboring Thailand. In 2019 alone, an estimated 1.1 million civilians were displaced from Myanmar, including 97,571 DPs identified by UNHCR as refugees or in ‘refugee-like’ situations in Thailand [6]. As is the case for the majority of the world’s displaced, DPs remain concentrated near the Thai-Myanmar border.

Displacement residential settings in Thailand comprise camps, but not exclusively. DPs also reside in remote villages and on employer-managed worksites, such as agricultural fields and temporary construction sites. These diverse displacement circumstances have emerged due to the Royal Thai Government (RTG)‘s ad hoc approach to asylum seekers. As a non-signatory to the 1951 Refugee Convention, it has generally treated them as temporary migrants by law, making them subject to arrest, detention, or deportation [7, 8]. The national response to chronic displacement from Myanmar has been particularly inconsistent. Fleeing persecution or effects of fighting (e.g., loss of land or livelihood) do not automatically qualify individuals for ‘displaced’ status and the UNHCR was actually banned from performing refugee status determination for asylum seekers from Myanmar in 2004 [9, 10]. Only select groups have been recognized as ‘displaced persons’ and granted ‘temporary shelters.’ Among DPs from Shan State, this included only one ‘temporary shelter’ in 2002 and still no guarantee of legal status (LS) determination. Furthermore, the RTG’s constitution only provides legal human rights protections to Thai citizens. Thus, the Thai-Myanmar border represents a geographic intersection point for displacement, precarious LS, and ethnicity-based and migration-based violence amid concentrated police and military surveillance [11]. Deprived of LS, permanent residency, or rights to legal work, DPs face compounded insecurity and HRVs in these precarious refuge settlements.

Women are especially vulnerable to HRVs in chronic displacement settings and perinatal health is particularly sensitive to related stressors and health care barriers. First, gender-based violence is typically exacerbated by conflict and displacement [12, 13]. Second, HRVs in work settings, public spaces, and healthcare facilities—realized or threatened—can prevent women from receiving timely and consistent reproductive health services, which are extremely important for preventing adverse perinatal outcomes [14–18]. Third, material deprivation, labor exploitation, and mobility restrictions—all psychologically, mentally and emotionally violent [2]—can affect maternal health and fetal growth during pregnancy by constraining maternal nutritional intake and exacerbating barriers to services [14, 15, 17–22]. Additionally, exposure to violence and mistreatment—acute or chronic—can pose psychosocial stress that adversely affects the intrauterine environment and can ultimately restrict fetal growth or induce pregnancy complications, including premature labor [23–31].

HRVs generally affect women’s reproductive health and perinatal outcomes adversely, yet varying degrees of impact in diverse contexts of chronic displacement remains largely unexplored. How do HRVs affect these outcomes when residential context, ethnicity, and migrant or refugee LS are considered? In other words, how sensitive are reproductive health outcomes to distinct and intersecting experiences of conflict-related violence and additional HRV threats in ‘post’-displacement settings, such as LS precarity, migrant worker exploitation, and ethnicity-based discrimination?

The current study uses novel survey data from the Thai-Myanmar border to investigate the pervasiveness of HRVs and their health implications among women recently pregnant, including Thai, Shan, and other ethnic minority groups who self-identify as either Thai, migrant, or refugee. The border with Shan State offers an informative study setting. Unlike more studied areas along the border, it is characterized by few factories and camps, along with many ethnic minority villages, agricultural fields and worksites, and informal refugee settlements—both recent and protracted. Within this diverse set of circumstances, there is a high concentration of personal displacement experiences.

Our study objectives are threefold. First, we document HRVs against women in a setting with relatively high proportions of chronically displaced people, specifically an international border of conflict where displaced persons reside both inside and outside unofficial, volatile camps. HRVs pertain to violations of personal safety, security, livelihoods, and health that are common to border areas and displacement situations. Second, we examine the social factors that are associated with HRV exposure and related marginalization including ethnicity, past displacement, LS, and living conditions. Third, we investigate how HRV frequency, timing, and type are linked to subsequent antenatal care use, pregnancy complications, and birth weight. Together, these aims elucidate the relationships between human rights and health, specifically how displacement and, ethnicity- and nativity-based marginalization shape perinatal health at international borders of conflict.

## Methods

### Data collection

The current study uses data from a larger cross-sectional survey conducted in 2016–2017 that addressed additional study objectives and consisted of two separate components: anthropometry and at-home interviews [11, 32]. Sample eligibility criteria included having been pregnant in the previous five years, living in one of the study sites, and having been between ages 15 and 35 years-old at the time of pregnancy and also at least 18 years-old at the time of the study.

The survey was undertaken in two selected sub-districts, chosen based on expert interviews with provincial hospital staff and community members to ensure adequate subgroup sizes for comparisons by maternal displacement experiences (e.g.*, *timing and circumstances) and current residential setting. The sub-districts comprised rural and semi-rural villages, where nearly all community members sought care at their designated district hospital and sub-district health center. This included migrants and DPs, who are eligible for public healthcare services regardless of LS. Women were recruited in two ways: by sub-district health center staff, based on recorded births and antenatal and immunization appointments, and by exhaustive referrals from village leaders and community volunteers in all accessible villages and worksites within the sub-districts, resulting in a total canvassing of the sites. The study aimed to enroll 800 participants to ensure that at least 600 would complete both survey components. With this goal, effective sample sizes and detectable differences were deemed achievable based on calculations completed before data collection (Table S2 online). All eligible women identified were recruited for participation both in each registered village within the two field sites and at worksites for which employer consent was obtained. Based on the sample composition, an additional set of neighboring communities from an adjacent sub-district (within 25 km) were included to enrich the sample with under-represented groups, specifically villages and worksites with very recent migrants. Ultimately, the study recruited participants from 37 registered villages, comprising 76 smaller communities or worksites.

Study participation was high (99%), with 824 eligible women successfully recruited. We attribute this to strong partnerships with local healthcare providers and community-based organizations working on social services and advocacy for marginalized groups in the study population. The response rate for questions used in the current study analysis was ultimately 70%, with 577 completed interviews.

Informed consent was obtained from all participants. Human subjects research was approved by the Chiang Mai Provincial Ministry of Public Health and the University of Wisconsin-Madison Institutional Review Boards. Given potential trauma and health needs uncovered in the study, all participants were given information for local mental health clinics and social work providers offering free patient counseling and public service assistance in the languages spoken by participants.

### Measures

All study data originated from questionnaire-assisted interviews conducted in respondents’ preferred languages by trained interviewers, following Thai and Shan scripts. Respondents were interviewed at their homes or their preferred location elsewhere and were reminded throughout the interview that they could skip any uncomfortable questions. Questions regarding HRVs were developed with local Shan researchers focusing on the types of violence and HRVs uncovered in prior ethnographic and interview-based research in the area [18, 33], local non-government organizational reports [34], and local focus groups and interviews [11, 32]. Common HRVs were physical violence, resource deprivation, labor exploitation or loss of livelihood, restricted mobility, and persecution. Questions developed in Thai, Shan, and English were cross-translated and pretested with non-study subjects for consistency [35]. English translations of all HRV questions are listed in the online supplementary materials.

The timing of the most recent occurrence for each specified event was classified relative to the end of each pregnancy (ever, ≤ 5 years prior, ≤ 1 year prior). Counts of any HRVs during these reference periods were categorized (0, 1, 2+), and separate binary indicators for each HRV type were generated (listed in online Table S1).

Primary pregnancy outcomes were antenatal care (ANC), complications, preterm birth (PTB), and birthweight. For each outcome, participants answered questions while referencing records in health facility-issued booklets. Unmet ANC need was based on responses to “how many times did you go in for prenatal care?” dichotomized into less than six visits or at least six visits, regardless of pregnancy duration [36]. Pregnancy complications were indicated by a “yes” response to any of following occurring during pregnancy, labor, or the puerperal period: high fever, hypertension, shock or seizure, premature rupture of membranes (> 24 h), premature labor, or hemorrhage. PTB (< 37 gestation weeks) was measured as a binary outcome, calculated from reported birthdates and due dates or, when unavailable, based on maternal report of PTB. Birth weight, read from the birth certificate at the time of the interview, was analyzed as a continuous variable.

Ethnicity and country of birth from self-report were coded as: ethnic Thai, ethnic Shan born in Thailand, other ethnic minority born in Thailand, Shan migrant, or other ethnic minority migrant. We created a single categorical variable for legal documentation based on the questions “Do you have a citizen card?” and (if no) “What type of identification card do you currently have?” Response categories were collapsed to reflect LS as follows: citizen card; legal resident card (also includes ‘legal alien’ card, which is most promising for future citizenship eligibility); passport or migrant worker permit; ‘unregistered resident’ paper; or no documentation. Each of the non-citizen LS categories represent some degree of LS precarity. Residence was self-reported and confirmed by the interviewer as town or village, worksite, or informal refugee camp.

Additional model covariates related to pregnancy risks [36] included: maternal age at birth (in years); maternal educational attainment (less than primary, primary complete, or secondary complete); and parity. Birthing facility and health insurance were considered but not included in models due to collinearity.

### Analysis

First, we measured and summarized HRV prevalence in the study sample. Second, to assess associations between social factors and HRV exposure we compared HRV prevalence between groups defined by country of birth, ethnicity, residence type, and LS, using robust standard errors to account for village clustering. Finally, to investigate how HRV exposures were linked to perinatal outcomes for index (most recent) pregnancies, we performed logistic regressions on antenatal care need, any pregnancy complications, and PTB as separate binary outcomes; and linear regressions on birth weight (grams) as a continuous outcome. We modeled the impact of HRV frequency (count) and type with separate models for different reference periods (ever, ≤ 5 years, and ≤ 1 year). Each model used generalized equation estimators that accounted for village clustering and adjusted for potential confounding factors, including maternal age, parity, and education.

As robustness checks of the HRV-outcome associations, we reran models adjusting for wealth. We additionally ran separate maternal fixed effects regressions on birth weight, using respondents’ recall of all past pregnancies, to assess the influence of unmeasured maternal factors.

All statistical analyses were performed in Stata 15. To reduce bias and maximize the use of available data, we used multiple imputation by chained equations with ten imputed datasets for all estimation models [37–39]. The following variables included imputed values (percent missing in parenthesis): birthweight (9%), occurrence of given type of HRV in last five years (1–2%), adequate ANC (10%), pregnancy complications (3%), preterm birth (3%), maternal country of birth (9%), ethnicity (5%), residence (3%), and LS (5%). In addition to the above variables, maternal language, village, and age were predictors in all imputation models.

## Results

Table 1 summarizes HRV prevalence and sample characteristics, with 42% of women reporting at least one HRV before or during their pregnancy and 25% reporting at least one HRV within the 5 years prior to the end of their pregnancy. Our study sample was diverse. Sixty-six percent were born in Myanmar and 89% did not identify as Thai. Twenty-nine percent were legal residents/aliens, 28% had at least some migrant documentation, and 20% either had ‘unregistered’ LS or no documents at all. Four percent were living in an unofficial refugee camp and 22% lived on worksites (93% agricultural orchards or fields; 5% factories; 2% other). Given the large proportion of respondents whose HRV experiences directly related to their displacement from Myanmar, including fleeing armed conflict, we also calculated the prevalence of HRVs related to deprivation and conflict-specific violence or persecution in Myanmar separately. Among women born in Myanmar, 47% reported at least one HRV in Myanmar, 24% reported water or food deprivation there, and 45% reported conflict-related violence or other persecution.

**Table 1:** Sample summary statistics with percentages or means (standard deviations)

	Total sample	Mother born in Thailand	Mother born in Myanmar
*n*	577	197	379
*Human Rights Violations*			
Any before pregnancy	43.7%	21.9%	53.8%
≤ 5 years before pregnancy	18.6%	5.9%	24.8%
*Ethnicity*			
Thai	9.2%	27.6%	0.7%
Shan	65.5%	36.6%	79.0%
Other ethnic minority group	25.4%	35.8%	20.4%
*Legal documentation*			
Thai citizen	15.1%	38.1%	4.3%
Legal resident/’alien’	29.3%	29.4%	29.3%
Migrant worker permit/ visa	28.4%	13.8%	17.1%
‘Unregistered resident' (10-year card) or no legal documentation	27.2%	17.7%%	31.0%
*Residence*			
Town/village	70.8%	84.1%	64.3%
Orchard, field, or other worksite	22.0%	12.3%	26.9%
Camp	7.2%	3.6%	8.7%
*Health and pregnancy*			
Antenatal care			
Any	89.4%	95.1%	86.7%
Timely	54.6%	59.8%	52.4%
Recommended visit frequency	61.7%	67.4%	59.1%
Hospital birth	85.6%	95.4%	80.8%
Any health insurance	81.5%	85.9%	79.2%
Pregnancy complications	18.8%	19.4%	18.0%
Preterm birth	17.7%	21.8%	15.6%
Birthweight			
Grams	2922 (463)	2937 (502)	2916 (440)
Low birth weight	13.1%	14.7%	12.0%
Maternal age at birth	24.9 (5.3)	23.3 (5.1)	25.6 (5.2)
Parity			
1	45.3%	54.7%	40.8%
2	37.5%	29.6%	41.3%
3+	17.1%	15.7%	17.9%

Table 2 further describes salient social determinants of HRVs by type and timing relative to pregnancy. As expected, prevalence of any HRVs was consistently lower for women who were Thai, born in Thailand, or both. Meanwhile, women in camps experienced the highest prevalence of any HRVs ever (83%), and a reduced prevalence of more recent HRVs (10 and 5% within 5 and 1 year of pregnancy). Other subgroups experienced less steep declines in more recent HRVs, with exposures remaining more elevated among participants without legal documentation or living on a worksite.

**Table 2:** Subgroup comparisons of HRV prevalence relative to end of pregnancy: ever and within one (≤ 1y) and five years (≤ 5y). *P*-values from F tests accounting for clustered standard errors are italicized

	Any HRV			Resource deprivation			Labor exploitation			Restricted mobility/ liberties			Violence/ Conflict		
	Ever	≤ 5y	≤ 1y	Ever	≤ 5y	≤ 1y	Ever	≤ 5y	≤ 1y	Ever	≤ 5y	≤ 1y	Ever	≤ 5y	≤ 1y
**Ethnicity**															
Thai	10%	4%	3%	5%	0%	0%	4%	1%	0%	1%	1%	1%	7%	2%	2%
Shan	52%	21%	11%	24%	4%	1%	17%	8%	4%	30%	11%	6%	38%	8%	3%
Other ethnic minority	32%	13%	8%	11%	5%	3%	14%	6%	3%	10%	8%	6%	15%	6%	2%
	*< 0.001*	*0.001*	*0.019*	*0.002*	*0.006*	*0.005*	*0.002*	*0.004*	*0.002*	*< 0.001*	*< 0.001*	*0.018*	*< 0.001*	*0.180*	*0.486*
**Country of birth**															
Thailand	19%	10%	4%	8%	2%	1%	8%	3%	1%	7%	4%	2%	8%	3%	1%
Myanmar	55%	21%	12%	24%	5%	2%	18%	8%	4%	30%	12%	7%	40%	9%	4%
	*< 0.001*	*0.005*	*0.004*	*< 0.001*	*0.012*	*0.131*	*< 0.001*	*0.046*	*0.061*	*< 0.001*	*0.012*	*0.009*	*< 0.001*	*0.005*	*0.016*
**Legal status**															
Citizen	10%	7%	5%	4%	2%	2%	4%	1%	0%	5%	4%	2%	4%	3%	2%
‘Alien resident’	51%	16%	8%	24%	2%	1%	17%	7%	4%	26%	8%	3%	31%	4%	1%
Passport or migrant permit	55%	25%	11%	26%	6%	2%	21%	9%	5%	29%	14%	7%	42%	12%	3%
10-year card	42%	9%	5%	13%	2%	0%	13%	6%	2%	20%	0%	1%	29%	2%	0%
Without documentation	49%	33%	30%	15%	6%	3%	15%	12%	6%	29%	22%	19%	32%	15%	9%
	*< 0.001*	*0.001*	0.0463	*< 0.001*	0.675	*0.805*	*< 0.001*	*< 0.001*	*< 0.001*	*< 0.001*	*< 0.001*	*0.002*	*< 0.001*	*0.052*	*0.148*
**Residential setting**															
Town/ village	36%	12%	5%	16%	2%	1%	13%	4%	2%	18%	6%	1%	25%	5%	1%
Worksite	54%	33%	22%	25%	8%	3%	20%	15%	9%	29%	19%	14%	36%	13%	5%
Camp	83%	10%	5%	31%	5%	0%	22%	0%	0%	34%	3%	0%	78%	7%	5%
	*< 0.001*	*0.001*	*< 0.001*	*< 0.001*	*0.012*	*0.002*	*0.233*	*< 0.001*	*< 0.001*	*0.001*	*0.014*	*< 0.001*	*< 0.001*	*0.240*	*0.108*

Resource deprivation was most common among women born in Myanmar and women with relatively more precarious LS. Women at worksites were most likely to have experienced resource deprivation ever (25%) and within five years of pregnancy (8%). Experiences of any labor exploitation before or during pregnancy were more common among women born in Myanmar, women without LS, and women living on worksites. Across reference periods, restricted mobility or liberties were most prevalent among women who were Shan, born in Myanmar, without LS or documentation, and living on a worksite. Violence or conflict-related HRVs were consistently most common among women who were ethnically Shan, born in Myanmar, unregistered or without legal documentation, or living in a camp or worksite.

Tables 3 and 4 summarize the associations between HRVs and pregnancy outcomes. Mothers experiencing two or more HRV’s ever, within 5 years, and within 1 year prior to pregnancy had significantly higher odds of unmet ANC need than mothers reporting no HRVs, with odds ratios (ORs) of 1.58 (90% Confidence Interval [CI]: 1.00, 2.49), 3.43 (90% CI: 1.68, 6.98), and 3.55 (90% CI: 1.45, 8.69) respectively (Table 3). Restricted mobility specifically was associated with higher odds of unmet ANC need (Table 4). Violence and conflict-related HRVs occurring within 5 years of pregnancy were also associated with higher odds of unmet ANC need. HRV count was not associated with higher odds of reported pregnancy complications (Table 3), but resource deprivation by itself was across all reference periods (Table 4). Although HRV count did not significantly predict PTB, it did significantly predict lower mean birth weight. HRVs specific to mobility and violence each predicted greater odds of PTB. Each type of HRV was also negatively associated with mean birth weight, although differences were only statistically significant for violent HRVs occurring within five years of pregnancy (Table 4).

**Table 3:** Adjusted odds ratios (ORs) and mean differences for pregnancy outcomes associated with HRV count exposures ever, ≤ 5 years (5y), and ≤ 1 year (1y) before the end of pregnancy. Confidence intervals (90%) in parentheses. Each model adjusts for maternal age, previous live birth, education, and country of birth

	Unmet ANC need (OR)			Any pregnancy complications (OR)			Preterm Birth (OR)			Birthweight (Difference in grams)		
HRV Count	Ever	≤ 5 years prior	≤ 1 year prior	Ever	≤ 5 years prior	≤ 1 year prior	Ever	≤ 5 years prior	≤ 1 year prior	Ever	≤ 5 years prior	≤ 1 year prior
0	Ref	Ref	Ref	Ref	Ref	Ref	Ref	Ref	Ref	Ref	Ref	Ref
1	0.56 *(0.27, 1.15)*	0.82 *(0.32, 2.12)*	0.89 *(0.52, 1.52)*	1.27 *(0.69, 2.34)*	1.03 *(0.62, 1.72)*	1.09 *(0.39, 3.03)*	0.59 *(0.40, 0.88)*	0.49 *(0.25, 0.93)*	1.26 *(0.39, 4.03)*	76 *(0, 151)*	− 50 *(− 186, 86)*	− 106 *(− 262, 50)*
2+	1.58 *(1.00, 2.49)*	3.43 *(1.68, 6.98)*	3.55 *(1.45, 8.69)*	0.90 *(0.55, 1.47)*	0.86 *(0.46, 1.62)*	0.82 *(0.32, 2.12)*	0.65 *(0.40, 1.05)*	1.39 *(0.88, 2.21)*	1.49 *(0.78, 2.83)*	−16 *(−83, 50)*	−151 *(− 275, −28)*	− 185 *(− 356, − 14)*

**Table 4:** Adjusted odds ratios (ORs) and mean differences for pregnancy outcomes associated with HRV exposures by type: ever, ≤ 5 years (5y), and ≤ 1 year (1y) before the end of pregnancy. Confidence intervals (90%) in parentheses. Each model adjusts for maternal age, parity (first birth), education, and country of birth

	Inadequate ANC Visits (OR)			Any pregnancy complications (OR)			Preterm birth (OR)			Birthweight (Difference in grams)		
HRV Type	Ever	≤ 5 years prior	≤ 1 year prior	Ever	≤ 5 years prior	≤ 1 year prior	Ever	≤ 5 years prior	≤ 1 year prior	Ever	≤ 5 years prior	≤ 1 year prior
**Resource deprivation**	1.19 *(0.80, 1.79)*	2.40 *(1.03, 5.58)*	1.39 *(0.34, 5.60)*	1.10 *(0.73, 1.64)*	3.07 *(1.34, 7.02)*	5.17 *(1.70, 15.75)*	1.16 *(0.69, 1.95)*	3.18 *(1.17, 8.63)*	3.37 *(0.82, 14.11)*	−20 *(−95, 55)*	− 138 *(−322, 47)*	− 127 *(− 411, 158)*
**Labor exploitation**	0.67 *(0.38, 1.20)*	1.59 *(0.80, 3.19)*	1.41 *(0.58, 3.40)*	1.61 *(1.04, 2.50)*	0.94 *(0.45, 1.96)*	0.75 *(0.27, 2.08)*	0.60 *(0.36, 1.01)*	0.67 *(0.30, 1.50)*	0.67 *(0.11, 4.15)*	−21 *(−98, 56)*	− 101 *(−233, 31)*	−67 *(− 234, 101)*
**Restricted mobility**	2.14 *(1.44, 3.17)*	1.86 *(1.02, 3.41)*	2.81 *(1.35, 5.85)*	0.78 *(0.49, 1.25)*	0.60 *(0.30, 1.19)*	0.86 *(0.32, 2.31)*	1.06 *(0.74, 1.51)*	1.08 *(0.59, 1.99)*	2.53 *(1.26, 5.07)*	−41 *(− 110, 28)*	−97 *(− 220, 27)*	− 179 *(− 363, 6)*
**Violence/ conflict**	1.29 *(0.87, 1.92)*	2.57 *(1.38, 4.81)*	1.74 *(0.79, 3.83)*	0.86 *(0.58, 1.28)*	1.36 *(0.68, 2.72)*	1.36 *(0.59, 3.17)*	0.92 *(0.62, 1.36)*	1.48 *(0.73, 3.00)*	3.55 *(1.48, 8.55)*	−24 *(−93, 44)*	− 166 *(− 299, −32)*	− 168 *(− 361, 24)*

When wealth is adjusted for, estimates do not substantially change (Table S3 online). Maternal fixed effects models revealed that experiencing two or more HRVs within five years or one year prior to pregnancy was consistently associated with a lower average birth weight compared to sibling births without the same HRV exposure (Table S4 online). Each HRV type was associated with lower average birthweight in within-mother comparisons, but standard errors were large.

## Discussion

We present a novel study of HRV prevalence and perinatal outcomes among women residing in a diverse context of chronic displacement at Thailand’s northern border with Myanmar. This is one of the most protracted displacement sites globally, where human rights abuses against DPs, migrant workers, and stateless persons have been consistently documented over decades, but often separately [40–53]. In this relatively small geographic space, women’s situations reflect broadly relevant experiences of protracted displacement, ethnic-based discrimination, and precarious LS and related threats to health.

Among study participants, 44% report at least one HRV in their lifetime. Ethnic minority women experienced disproportionately high HRV prevalence generally, particularly women who had migrated to worksites or experienced chronic displacement elsewhere. Women in the study who were born in Myanmar reported similar HRV frequencies to women still living in conflict zones within Myanmar in 2009 [33], suggesting their experiences and histories reflected the general situations in Myanmar from which people have fled over the prior decade. Compared to other studies that have measured HRVs among migrants in Thailand, the HRVs we documented were similar in type but higher in prevalence, possibly because our study included more forced migrants in addition to migrant workers [40, 54].

The HRVs documented on the Thai side of the border are not unique to this study. Similar reports document abuses and fears experienced by migrants in other parts of Thailand and other international border regions in recent decades [41, 42, 45–51]. Experiences of arrest, deportation, and related threats by law enforcement—along with debt, labor exploitation, and trafficking threats introduced by employers and brokers—all contribute to chronic stressors, adverse mental health, and repeated HRVs [40, 41]. Furthermore, past studies document how migrant women experience greater violence and HRVs than men at the border [42]. Beyond past studies that have focused more exclusively on recent DPs or migrant workers in larger industrial work sites, this study further reveals how violence against women pervades other settings. It is the first study to assess HRVs at the Shan State border and across multiple migrant and DP settings, including sites of chronic and intergenerational displacement, such as camps (‘temporary shelters’), agricultural work sites, and other general residential settings.

Findings highlight unique vulnerabilities of DPs and more generalized LS-related vulnerabilities. Women recognized by the RTG as temporarily displaced with 10-year resident cards and living in a camp appear to be buffered from ongoing HRVs, relative to other migrants. However, despite this place-based protection, other costs are incurred. During separate fieldwork interviews, residents expressed lacking the freedom to safely leave and work—rights not fully captured in the responses analyzed in the current study. Over the two decades since it was established, more camp residents have left voluntarily due to diminishing aid and inability to work. Although, separated from camps, findings show DPs face other acute HRV risks associated with precarious LS, comparable to vulnerable migrant workers and stateless persons [6]. Women without documentation experienced the greatest HRV burden immediately preceding pregnancies.

Our study is one of the first to link perinatal outcomes to multiple types of HRVs associated with ethnic discrimination, displacement, and militarized borders. Exposures to HRVs within the prior five years were associated with lower ANC use, more pregnancy complications, and lower birth weight. The strength of these associations depended on the number of HRVs and, in some cases, the type. Absolute magnitudes of associations were comparable to health advantages attributed to maternal education—one of the strongest predictors of women’s health and birth outcomes globally [55–57]. The reduction in birth weight associated with experiencing two or more HRVs in the preceding five years (− 151 g) was comparable to the weight increase associated with mothers finishing secondary school, relative to never attending school (197 g). The birth weight association was robust to additional adjustments for wealth and preterm labor, suggesting potential psychosocial or nutritional mechanisms causing restricted intrauterine growth. We did not find associations between overtly violent HRVs and all perinatal outcomes to be as strong as expected, which may have been due to under-reported adverse outcomes or survival bias. One other study at the border uncovered a significant association between pregnancy complication symptoms and similar exposures to conflict violence, as well as intimate partner violence [43].

Future research is needed to further investigate how to improve ANC accessibility. Based on survey interviews and related field work, and also due to Thailand’s universal health care coverage plan, we do not interpret differences in unmet ANC need as indicative of direct legal barriers to health services. Instead, we attribute differences to other barriers closely linked to LS and HRVs, particularly transportation needs, necessary work leave, potential language barriers, discrimination or stigma at health facilities, or fear of arrest or deportation on the way to a facility. More research could also inform efforts to further tailor health care and prevention to specific HRV-related needs, including nutritional and psychosocial needs potentially underlying lower birth weight.

To improve existing healthcare delivery, HRV-related questions could be incorporated into health screenings. Additionally, existing community-based organizations that help women access Thailand’s universal healthcare coverage plan could be scaled to reach more women living on worksites and others with precarious LS. Specific actions include expanding home visits and safe public or health facility-sponsored transportation to care centers. This would require workplaces to allow for daytime healthcare visits and authorities running ensure that border control and related LS surveillance to not block routes to facilities or otherwise interfere with healthcare delivery directly or through intimidation. Finally, the future of population health at the Thai-Myanmar border, and other similar international border settings, ultimately depends on more structural responses that universally protect human rights for DPs, migrants, and other marginalized ethnic minorities. This requires more inclusive refugee and citizenship laws for LS determination in Thailand, including wider allowance for the UNHCR to conduct refugee status determination. Similar LS provisions are necessary for migrants with precarious LS and stateless individuals, likely through coordinated efforts by UNHCR and IOM. Second, human rights protections and enforcement must be universal, i.e.*,* regardless of legal documentation or migrant status, which requires greater transparency and cooperation with the UN and other international human rights agencies.

## Limitations

The current study is limited by its cross-sectional design, which cannot entirely disentangle causal associations and underlying mechanisms linking HRVs to perinatal outcomes. Despite high participation, we were unable to sample a nontrivial number of women who were highly mobile or living in unreachable worksites. Women in these missing situations may have experienced more HRVs and more adverse perinatal health outcomes. Recall bias may have also contributed to measurement error, particularly for PTB and pregnancy complications, which were more likely to be missing from participants’ birth records. Many of the potential biases introduced by selection and measurement error would lead to underestimations of these associations. For instance, maternal and child survival both likely biased estimates toward the null due to undetected and unreported pregnancy losses, maternal deaths, and potentially lower fertility among populations disproportionately affected by severe HRVs. Women who had faced the most severe conflict-related HRVs in Myanmar were probably under-represented, as they are less likely to have survived and moved to Thailand.

## Conclusions

Human rights violations are common experiences for women living at the Thai-Myanmar border, particularly in settings affected by displacement. These experiences reflect historical violence in Myanmar, as well as unsafe working conditions, precarious LS, and other security threats faced by DPs and other ethnic minorities in Thailand. Women who experienced HRVs close to pregnancy experienced greater unmet healthcare need and lower infant birth weights. Furthermore, HRV frequency over the life course of women generally carried enduring associations with perinatal outcomes. Future work is needed to determine the most effective ways to deliver timely and safe perinatal health care. Thailand’s public health infrastructure and other local organizations could be expanded to enhance existing community-based health care, including HRV screenings to detect related perinatal risks early. To be successful and equitable, the public health system must also remain sovereign from border policing and related LS surveillance. Policy change is additionally critical to structurally ameliorate protracted displacement and precarious LS situations at the border, including statelessness and protracted ‘temporary’ statuses and shelters.

## Supplementary Information

**Additional file 1.**

## Data Availability

The data that support the findings of this study are available on request from the corresponding author SK. Data are not publicly available due to them containing information that could compromise research participant privacy.
