# Supplementary material for: Reproductive health at conflict borders: a cross-sectional survey of human rights violations and perinatal outcomes at the Thai-Myanmar border
Source: Confl Health. 2021 Mar 10;15:15. doi: 10.1186/s13031-021-00347-8 (PMC7945312; doi:10.1186/s13031-021-00347-8)
Supplement: Supplementary file 1 — Additional file 1. [file 13031_2021_347_MOESM1_ESM.pdf]

ONLINE SUPPLEMENTARY FILE

Table S1. Human rights violation questions and categorizations.
Table S2. Sample Size Calculations.
Table S3. Adjustments for wealth
Table S4. Maternal fixed effects model results.

Table S1. Human rights violation questions and categorizations. Human Rights Violation Categorizations of original survey questions, based on Universal Declaration of Human Rights

Resource/Property Deprivation (Article 17, Article 25):
Have you ever been in a situation in which you lacked food or clean drinking water?
Have you ever slept outside or in the forest because you did not have anywhere else to sleep?
Have you ever been in pain or ill and not receive care or medicine for it?
Did you ever have any of your belongings seized or destroyed, such as food, livestock, or crops?

Labor Exploitation (or Loss of Livelihood) (Article 23):
While in Thailand, has your family ever been without work long enough that you did not have enough to eat or did not have a safe place to live?
Have you ever worked but did not receive your pay as originally agreed upon?
Have you ever worked with an employer who refused to give you the necessary documents for a work permit?
Have you ever felt taken advantage of by a middleman/ broker?
Have you ever had an employer or broker that you feared would harm you?
Have you ever feared an employer or broker would touch you in a way you don't want to be touched?

Violence & Conflict (Multiple articles):
Have you ever had to flee your village because of conflict or because your village was destroyed?
Have you ever had a partner die a violent death?
Did you ever witness a temple or other religious monument of importance to you destroyed or desecrated?
Did you ever witness a Shan military force come into your village?
Did you ever witness a Burmese military force come into your village?
Were you ever forced to work for soldiers?
Was anyone in your family ever forced to serve in combat by military forces?
Did you ever see an explosive go off?
Did you ever witness fighting or homes being destroyed?
Did you ever witness a corpse of someone who died in battle?
Was your home ever raided by soldiers?
Did anyone in your family ever get kidnapped, get taken by the military, or disappear?
Did you ever witness someone being severely beaten, raped, or killed?
Were you ever bound up?
Did you ever witness a violent event in Burma that gave you nightmares?
Were you ever scared that someone would take your life?

Limited mobility or unlawful Persecution (Articles 13 & 9):
Have you ever hid in order to avoid being arrested?
Have you ever been arrested?
Has anyone else in your family been arrested?
Have you ever felt afraid or scared when traveling outside of your village/neighborhood?
Have you ever avoided traveling outside of your village/neighborhood out of fear?

Table S2. Sample Size Calculations.

The study sampling approach was finalized based on sample size calculations to detect differences in birth weight between mothers based on ethnicity, country of birth, and legal status. Implicit in these comparisons are substantial differences in exposure to violence, including human rights violations in Thailand, Myanmar, or both.

Below we present our calculations of detectable differences completed before data collection, based on a two-sided significance level of 0.05 and 80% power and using the following formula(1):

$$\Delta = (1.96+0.84)*(\text{projected standard error})$$

In order to take into account adjustment for other variables correlated with the outcome of interest, we used "effective sample size": $n*\sqrt{(1-R^2)}$, which assumed other variables were correlated with the variables of interest at $R^2=0.2$, for Δ calculations.

No complete sampling frame existed for our population of interest. We expected to identify at least 800 eligible study participants based on provincial Ministry of Public Health Records and interviews with local village leaders and community-based organizations working directly with groups underrepresented in public health records, including migrant workers and individuals with precarious legal status(2). We aimed to recruit 800 participants, with the expectation of approximately 75% retention for two data collection steps, anthropometric measurements conducted at healthcare centers and village centers (not included in current study's scope) and questionnaire-based interviews typically completed at respondents' homes. Based on expert interviews, we anticipated a relatively even distribution of participants across the following comparison groups: Thai, Shan born in Thailand, other ethnic minorities born in Thailand, Shan born in Myanmar, and other ethnic minorities born in Myanmar. Precarious legal status was understood to be relatively common among all ethnic minority groups, including individuals born in or outside of Thailand. We calculated detectable differences in birth weight based on expected subgroup sample sizes of 100.

Table S2a. Effective sample size and standard error calculations, based on estimated subgroup sample sizes(2) and birth weight distributions reported for Thai births(3) and ethnic minority births at the border(4) during the study period.

	Subgroup n	Effective n	Estimated mean birth weight in grams	Estimated σ	Standard error (σ/√n)
Thai	100	89	3237	412	44
Minority subgroups (4 total)	100	89	2950	500	53

Table S2b. Detectable difference calculations for subgroup birth weight comparisons.

	Comparing Thai and minority participants (each n=100)	Comparing between minority subgroups (each n=100)
Standard error ($\sqrt{((\sigma_1^2/n_1)+(\sigma_2^2/n_2))}$)	66	70
Δ (grams)	185	197
Δ (in standard deviation units)	0.40	0.42

As shown above, we expected to be able to detect subgroup birth weight differences between 185 and 197 grams, or 0.40 and 0.42 standard deviation units. We considered these to be reasonable based on past studies in these study populations, suggesting a difference of 287 grams between birth to ethnic Thai mothers and to ethnic minority women at the Thai-Myanmar border(3, 4). These estimates were based on limited data availability and thus were only approximate. Specific limitations include no adjustment for clustering within sub-district or village. However, these were taken into account in the final regression analyses.

Table S3. Adjustments for Wealth. Robustness checks were run with the perinatal outcome models adjusting for wealth, based on a battery of questions related to assets. Respondents were asked 18 (yes/no) questions regarding home ownership and household belongings. We computed interitem correlations using the full set of 18 questions and included the 9 indicators from this list that achieved an item-rest correlation of ≥ 0.3. This cut-off was chosen to achieve a final assets scale of standardized item scores with a Cronbach's alpha of 0.8 (mean=-0.1, standard deviation=0.7). The nine binary indicators included home ownership and the following household belongings: car/truck, motorcycle, radio, phone, satellite, refrigerator, rice cooker, and fan.

Table S3a. Adjusted odds ratios (OR's) and mean differences for pregnancy outcomes associated with HRV count exposures ever, ≤ 5 years (5y), and ≤ 1 year (1y) before the end of pregnancy. Confidence intervals (90%) in parentheses. Each model adjusts for maternal age, parity (first birth), education, country of birth, and household assets.

	**Inadequate ANC Visits (OR)**			**Any pregnancy complications (OR)**			**Preterm birth (OR)**			**Birthweight (Difference in grams)**		
**HRV Count**	**Ever**	**≤ 5 years prior**	**≤ 1 year prior**	**Ever**	**≤ 5 years prior**	**≤ 1 year prior**	**Ever**	**≤ 5 years prior**	**≤ 1 year prior**	**Ever**	**≤ 5 years prior**	**≤ 1 year prior**
0	Ref	Ref	Ref	Ref	Ref	Ref	Ref	Ref	Ref	Ref	Ref	Ref
1	0.49 *(0.24, 0.99)*	0.75 *(0.32, 1.75)*	0.76 *(0.49, 1.17)*	1.21 *(0.69, 2.14)*	1.00 *(0.59, 1.67)*	1.01 *(0.37, 2.75)*	0.67 *(0.43, 1.04)*	0.54 *(0.27, 1.09)*	1.56 *(0.53, 4.63)*	81 *(6, 157)*	-48 *(-178, 81)*	-104 *(-255, 47)*
2+	1.47 *(0.95, 2.28)*	2.84 *(1.46, 5.51)*	2.78 *(1.20, 6.45)*	0.86 *(0.54, 1.38)*	0.76 *(0.41, 1.42)*	0.69 *(0.29, 1.66)*	0.70 *(0.43, 1.15)*	1.76 *(1.11, 2.79)*	2.06 *(1.10, 3.86)*	-12 *(-77, 52)*	-180 *(-345, -14)*	-180 *(-345, -14)*

Table S3b. (Included at end of file due to size). Adjusted odds ratios (OR's) and mean differences for pregnancy outcomes associated with HRV exposures by type: ever, ≤ 5 years (5y), and ≤ 1 year (1y) before the end of pregnancy. Confidence intervals (90%) in parentheses. Each model adjusts for maternal age, parity (first birth), education, country of birth, and household assets.

Table S4. Maternal fixed effects model results. Adjusted mean birth weight differences (grams) between sample births (n=1,185) associated with maternal HRVs within 5- and 1-year windows before and during pregnancy. Confidence intervals (90%) are in parentheses. Each model adjusts for maternal age, parity (first birth), and birth year.

Human Rights Violations	Birthweight (Difference in grams)	
	≤ 5 years	≤ 1 year
Total count		
0	(Ref)	(Ref)
1	1	-11
	(-204, 206)	*(-251, 229)*
2+	-137	-125
	(-310, 36)	*(-396, 146)*
Type		
Resource deprivation	-37	-54
	(-349, 275)	*(434, 326)*
Labor exploitation	-15	-185
	(-275, 245)	*(-497, 127)*
Restricted mobility	26	-22
	(-199, 251)	*(-310, 266)*
Violence/ conflict	-182	-54
	(-460, 96)	*(-342, 234)*

Table S3b. Adjusted odds ratios (OR's) and mean differences for pregnancy outcomes associated with HRV exposures by type: ever, ≤ 5 years (5y), and ≤ 1 year (1y) before the end of pregnancy. Confidence intervals (90%) in parentheses. Each model adjusts for maternal age, parity (first birth), education, country of birth, and household assets.

	**Inadequate ANC Visits (OR)**			**Any pregnancy complications (OR)**			**Preterm Birth (OR)**			**Birthweight (Difference in grams)**		
**HRV Type**	**Ever**	**≤ 5 years prior**	**≤ 1 year prior**	**Ever**	**≤ 5 years prior**	**≤ 1 year prior**	**Ever**	**≤ 5 years prior**	**≤ 1 year prior**	**Ever**	**≤ 5 years prior**	**≤ 1 year prior**
**Resource deprivation**	1.14 *(0.79, 1.64)*	1.99 *(0.85, 4.67)*	1.08 *(0.24, 4.91)*	1.07 *(0.71, 1.62)*	2.86 *(1.24, 6.57)*	4.74 *(1.56, 14.39)*	1.26 *(0.73, 2.16)*	4.14 *(1.78, 9.62)*	4.31 *(1.18, 15.69)*	-17 *(-91, 57)*	-129 *(-308, 51)*	-115 *(-398, 168)*
**Labor exploitation**	0.64 *(0.34, 1.22)*	1.43 *(0.77, 2.67)*	1.30 *(0.59, 2.87)*	1.60 *(1.05, 2.44)*	0.90 *(0.44, 1.82)*	0.73 *(0.27, 1.93)*	0.63 *(0.38, 1.04)*	0.76 *(0.32, 1.79)*	0.72 *(0.12, 4.51)*	-19 *(-97, 58)*	-96 *(-221, 29)*	-63 *(-229, 103)*
**Restricted mobility**	2.06 *(1.41, 3.00)*	1.62 *(0.96, 2.76)*	2.16 *(1.06, 4.41)*	0.76 *(0.48, 1.19)*	0.55 *(0.29, 1.03)*	0.74 *(0.30, 1.82)*	1.12 *(0.75, 1.69)*	1.26 *(0.64, 2.46)*	3.96 *(2.28, 6.89)*	-39 *(-106, 29)*	-91 *(-203, 21)*	-173 *(-343, -2)*
**Violence/ conflict**	1.19 *(0.77, 1.83)*	2.20 *(1.19, 4.05)*	1.44 *(0.64, 3.23)*	0.82 *(0.55, 1.22)*	1.26 *(0.65, 2.42)*	1.23 *(0.54, 2.82)*	1.01 *(0.67, 1.53)*	1.88 *(0.95, 3.74)*	4.54 *(1.79, 11.46)*	-21 *(-87, 45)*	-160 *(-294, -26)*	-161 *(-359, 38)*

REFERENCES

1. Gelman A, Hill J. Sample size and power calculations. In: Gelman A, Hill J, editors. Data Analysis Using Regression and Multilevel/Hierarchical Models. Analytical Methods for Social Research. Cambridge: Cambridge University Press; 2006. p. 437-56.
2. Koning SM. Structural Violence, Life Stressors and Maternal and Child Health at the Thai-Myanmar Border: The University of Wisconsin-Madison; 2018.
3. Mikolajczyk RT, Zhang J, Betran AP, Souza JP, Mori R, Gülmezoglu AM, et al. A global reference for fetal-weight and birthweight percentiles. The Lancet. 2011;377(9780):1855-61.
4. Turner C, Carrara V, Thien NAM, Paw NMK, Rijken M, McGready R, et al. Changes in the body weight of term infants, born in the tropics, during the first seven days of life. BMC Pediatr. 2013;13(1):1-4.
